# The pathological structure of the perivascular niche in different microvascular patterns of glioblastoma

**DOI:** 10.1371/journal.pone.0182183

**Published:** 2017-08-03

**Authors:** Jintao Chen, Sifeng Mao, Haifang Li, Mingcheng Zheng, Linglu Yi, Jin-Ming Lin, Zhi-xiong Lin

**Affiliations:** 1 Department of Neurosurgery, The First Affiliated Hospital of Fujian Medical University, Fuzhou, Fujian, China; 2 Department of Chemistry, Beijing Key Laboratory of Microanalytical Methods and Instrumentation, Tsinghua University, Beijing, China; 3 Department of Neurosurgery, Beijing Sanbo Brain Hospital, Capital Medical University, Beijing, China; Universita degli Studi di Bari Aldo Moro, ITALY

## Abstract

The perivascular niche is critical for intercellular communication between resident cell types in glioblastoma (GBM), and it plays a vital role in maintaining the glioma stem cell (GSC) microenvironment. It is shown in abundant research that different microvascular patterns exist in GBM; and it can be implied that different microvascular patterns are associated with different pathological structures in the perivascular niche. However, the pathological structure of the perivascular niche is still not clear. Here, we investigated the distribution and biological characteristics of different microvascular pattern niches (MVPNs) in GBM by detecting the expression of CD34, CD133, Nestin, α-SMA, GFAP and CD14 in the perivascular niche using multiple -fluorescence. The four basic microvascular patterns are microvascular sprouting (MS), vascular cluster (VC), vascular garland (VG), and glomeruloid vascular proliferation (GVP). By analyzing the proportion of the area of each marker in four types of formations, the results indicated that the expression of CD34, CD133 and Nestin in MS and VC was significantly lower than that in VG and GVP (P<0.05). Furthermore, the results showed that α-SMA expression different in the MS, VC, VG and GVP (P<0.05). However, the expression of GFAP and CD14 in each type of formation exhibited no significant difference (P>0.05). According to the area distributions of different markers, we mapped four precise simulation diagrams to provide an effective foundation for the accurate simulation of glioblastoma in vitro.

## Introduction

Glioma is the most common primary tumor of the adult central nervous system. The incidence of glioma ranks in the top 10 of human tumors. Glioma patients have a poor clinical outcome, especially those with primary glioblastoma; the average survival time of most patients is only 12 to 15 months after diagnosis [1]. Malignant glioblastoma exhibits a high self-renewal and proliferation capacity of glioma stem cells and the perivascular niche that it relies for existence is considered a root cause of drug resistance and recurrence for glioblastoma treatment [2]. Therefore, studying the interactions of the perivascular niche and its surrounding cells is of great value in unraveling the mechanisms of drug resistance in malignant gliomas and exploring new therapeutic approaches [3,4]. However, a great deal of the research is only about discussing the relationship between glioma stem cells and endothelial cells [5], and still restricted to researching in-vitro culture of two kinds of cells or multiple cells without accurate simulation [6]. Therefore, an understanding of the components and structure of the perivascular niche in glioblastoma is particularly important for designing in vitro and in vivo GBM tumor models [7,8].

Glioblastoma multiforme remains the most common primary malignant brain tumor in adults, accounting for more than 50% of primary malignant gliomas [9]. Despite new therapeutic strategies that are more rich and findings that indicate that most drugs perform well in experiments [10,11], in recent years, the clinical outcome of primary glioblastoma has remained poor [12]. Upon investigating the reasons for the large differences between the experimental results and clinical trials, the tumor structure and pathogenetic mechanisms are still largely unknown. At present, many studies are limited to mixed cultures of two or more cell types [13–15] in vitro, and the use of static cell culture is not comparable to the real niche of glioma stem cells (GSCs) that are endowed with a self-renewal ability and a multi-potent nature [16,17]. Therefore, we urgently need a new microenvironment that can effectively simulate different cells and proportions that could provide the basis for research on subsequent cellular interactions. Recently, two complementary niches have been described in GBM [18]. The first one is a perivascular niche composed of vessels, GSCs, pericytes, and tissue specific components. The second one is a hypoxic niche that is found in regions with low oxygen tension, such as the core of a tumor.

Angiogenesis is a prominent feature of primary glioblastoma [19]. The abundance of abnormal vasculature not only supplies oxygen and nutrients, but also facilitates glioblastoma recurrence, proliferation and invasion. Anti-angiogenic represents a promising therapeutic target. However, the development of diverse resistance mechanisms lessens the therapeutic effect of anti-angiogenic treatments. For this reason, our group, in accordance with recent studies, has localized CSCs in this disease adjacent to endothelial cells (EC) in what has been termed a perivascular niche, spurring investigation into the role of EC—CSC interactions in glioblastoma multiforme treatment resistance and the root cause of recurrence [2]. Research by our team and many other scholars demonstrated that CD34 positive blood vessels can be divided into four different MVPs on the basis of four different vascular niche pathologic structures [20]. In this article, we examined continuous formalin-fixed paraffin-embedded tissue sections by using quad-immunofluorescence histochemical staining for glioblastoma and classified heterogenous microvascular features. The observations and statistics about the structural characteristics of the perivascular niche in the four MVPs provide the basis for in vitro simulation.

## Materials and methods

### Patients and tissue samples

This study utilized samples from patients with primary glioblastoma who underwent surgery at the Department of Neurosurgery, Beijing Sanbo Brain Hospital of Capital Medical University between January 2014 and June 2015. Glioblastoma patients with recurrent disease or without incomplete medical records were excluded. Finally, 61 patients were enrolled. Informed consent was obtained from all patients according to the research proposal approved by the local ethics committee of the Beijing Sanbo Brain Hospital. Tumor specimens were reviewed and evaluated by two experienced neuropathologists who were blinded to the study, using the principles of WHO classification for tumors in the central nervous system [9]. Clinical information was obtained by reviewing the medical records (Table 1).

**Table 1:** Clinical characteristics of patients.

Characteristics		n = 61
Gender	Male/ Female	30/31
Age (years)	Median/ Range	53.04/17-73
KPS	≤70/ >70	21/40
Resection degree	Subtotal/ Total	9/52
Radiotherapy	Yes/No	38/23
Chemotherapy	For ≥6 cycles/<6 cycles	41/20

Abbreviations: KPS, Karnofsky Performance Scale.

### Immunofluorescence staining

Sections were fixed in 10% Neutral Buffered Formalin (NBF), processed with the Leica ASP6025 tissue processor (Leica Microsystems, Germany), embedded in paraffin and cut into several continuous sections at a thickness of 4 μm. Sections were deparaffinized in xylene, dehydrated in graded alcohol and conditioned. Antigens were retrieved with All-purpose Powerful Antigen Retrieval Solution (1:10, Beyotime, P0088, China). Slides were blocked for 30 minutes with QuickBlock^™^ Blocking Buffer (Beyotime, P0220, China). Primary antibodies were applied at optimized concentrations previously determined on control tissues, and the following primary antibodies were used: anti-Nestin (1:150, Abcam, ab22035, UK), anti-CD133 (1:100, biorbyt, orb99113, UK), anti-CD34 antibody (1:100, Santa Cruz, SC-7045, USA), anti-α-SMA (1:200, Abcam, ab5694, UK), anti-CD14 (1:100, Abcam, ab133335, UK), and anti-GFAP (1:100, ZSGB-BIO, ZM-0118, China). The sections were incubated with the diluted primary antibodies. Then, the sections were incubated with secondary antibody solution (Abcam, UK): Alexa Fluor^®^ 488 (Donkey Anti-Goat, ab150129), Alexa Fluor^®^ 555 (Donkey Anti-Mouse, ab150106), Alexa Fluor^®^ 647 (Donkey Anti-Rabbit, ab150075), which were prepared according to the manufacturer’s instructions with predetermined dilutions. Slides were counterstained with DAPI (4‘,6-diamidino-2-phenylindole Beyotime C1002, China) at 5 mg/ml in PBS for 10 minutes, mounted with Antifade Mounting Medium and kept at -20°C. The negative control sections were incubated with secondary Abs in PBS omit the primary antibodies, and known positive tissue sections (human osteosarcoma cells for Nestin and CD133; human lung carcinoma tissue for CD34, α-SMA and CD14; and normal brain tissue from patients with brain trauma for GFAP) were used as positive control.

The stainings were performed consecutively. The operation for each marker was completed before the application of the next antibody. For example, triple stainings (CD34, CD133, and Nestin), the sections were first incubated with the goat anti-human CD34 for 4°C overnight (approximately 16 h). Followed next day wash slides 3 times with PBS and visualized with Alexa Fluor^®^ 488 incubate at room temperature for 60 min in the dark. Wash slides 3 times with PBS and apply serum blocking buffer for 30 min at room temperature. Shake off normal serum and apply mouse anti-Nestin antibody for 2 h at room temperature. Then, wash the antibody and apply Alexa Fluor^®^ 555 at room temperature for 60min. With the above method, after serum blocking the slides apply the rabbit anti-CD133 antibody incubate at room temperature for 2 h. Repeat wash the antibody and apply Alexa Fluor^®^ 647 at room temperature for 60min. In all cases, the slides were counterstained with DAPI and finally cover slipped.

### Evaluation of staining

Fluorescent images were obtained with a high objective on a Zeiss LSM780 motorized microscope (Zeiss Microsystems, Germany) with a high sensitivity phosphorus gallium arsenide (GaAsP) detector. Images were obtained by sequentially scanning each channel with specific pairs of highly selective custom-made filters to eliminate the crosstalk of the fluorophores and ensure a reliable quantification. To guarantee the reliability, sections in the experiment were stained with multiple immunofluorescence markers, and images were captured under the same exposure settings at a 1024×1024 pixel resolution. Because the antibody signal areas and intensities were highly variable in the tissues, and in consideration of the uneven distribution of microvessels in tumor tissues, we choose a 2-step “multi-hotspot” assessment as described by Chen et al. [20]. The number of positive endothelial or tumor cells was counted at 200× magnification (0.18 mm^2^ per field size). Five hotspot microvessels fields were selected for each tumor specimen and were evaluated by two trained investigators who were unaware of the pathological diagnosis and clinical data; the investigators analyzed multiple stainings and classified microvascular patterns.

### Statistical analysis

All images of 61 primary glioblastoma were transferred to proprietary format and were then subjected to further processing and analysis using Image-Pro Plus for Windows, version 6.0 (Media Cybernetics, Silver Spring, MD). The program was calibrated with a standard scale according to the directions. To calculate the area occupied by immunolabeling, cells were semi-quantitatively reported as square microns per 20× objective area, utilizing an HSI-prole threshold for each of the channel signals and for all slides analyzed. The proportion of each positive area was calculated by using the following formula: positive area = (positive area/examination area) × 100.

All data were analyzed using the SPSS 20.0 software. Differences among the four microvascular pattern groups were investigated using Student’s t test and ANOVA analysis. A P value < 0.05 was considered statistically significant.

## Results

### Determination of microvascular patterns

**Fig 1:**
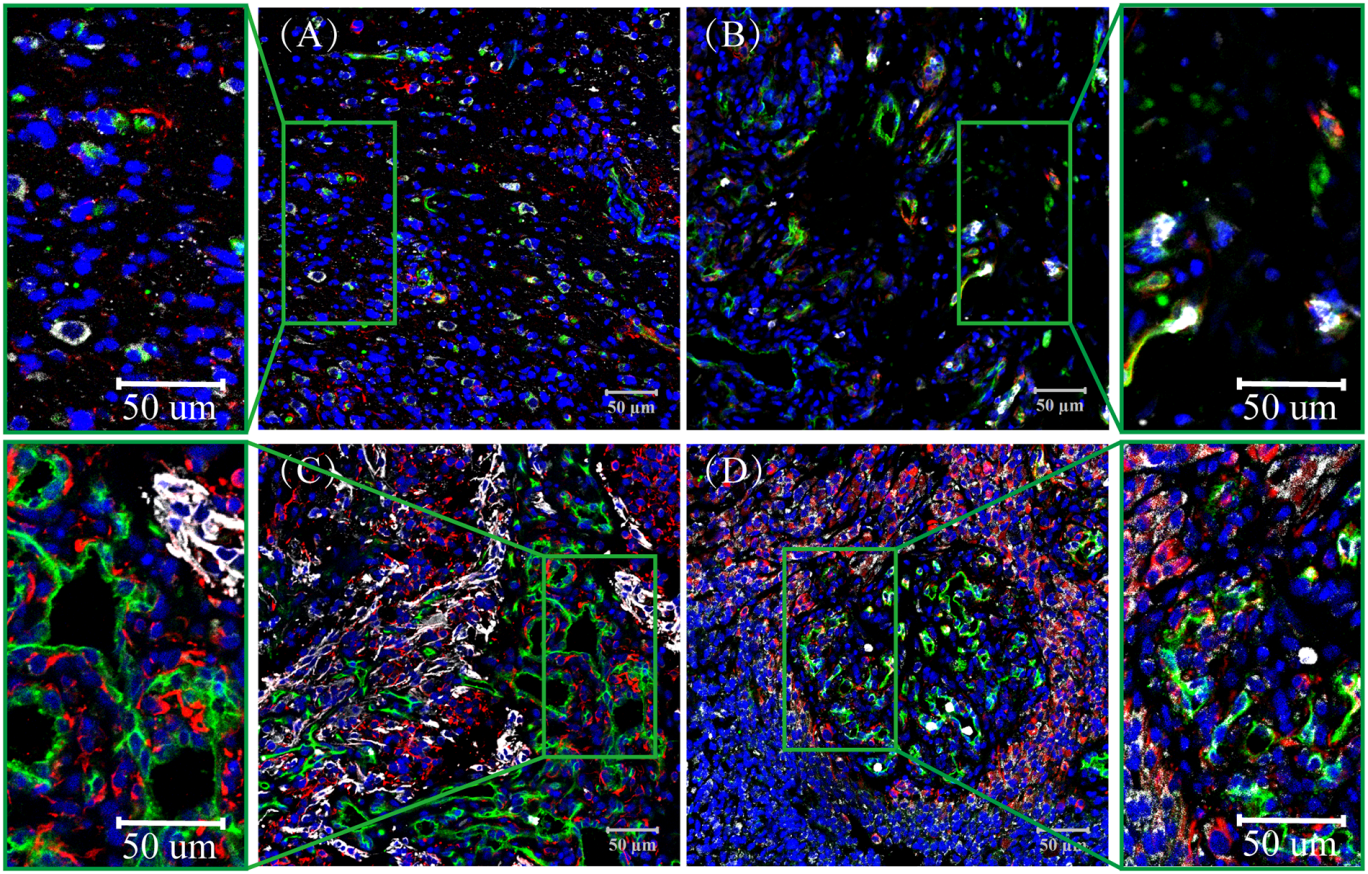
CD133/Nestin/CD34 Expression in Glioblastoma, CD133 (Purple), Nestin (Red), CD34 (Green). Nuclei are Counter stained with DAPI (Blue). (A) microvascular sprouting (MS), many Nestin and CD133 positive cells are scattered; (B) vascular cluster (VC), the Nestin and CD133 positive cells have a regional maldistribution; (C) vascular garland (VG), a mass of Nestin positive cells are accumulated as a cluster. Nestin was expressed not only on GSCs but also in the brain capillaries on endothelial cells, and CD133 positive cells accumulated around the blood vessels; (D) glomeruloid vascular proliferation (GVP), with a large number of Nestin and CD133 positive cells in the periphery of vessels and fewer in the avascular area. Nuclei are always counterstained with DAPI (Blue).

**Fig 2:**
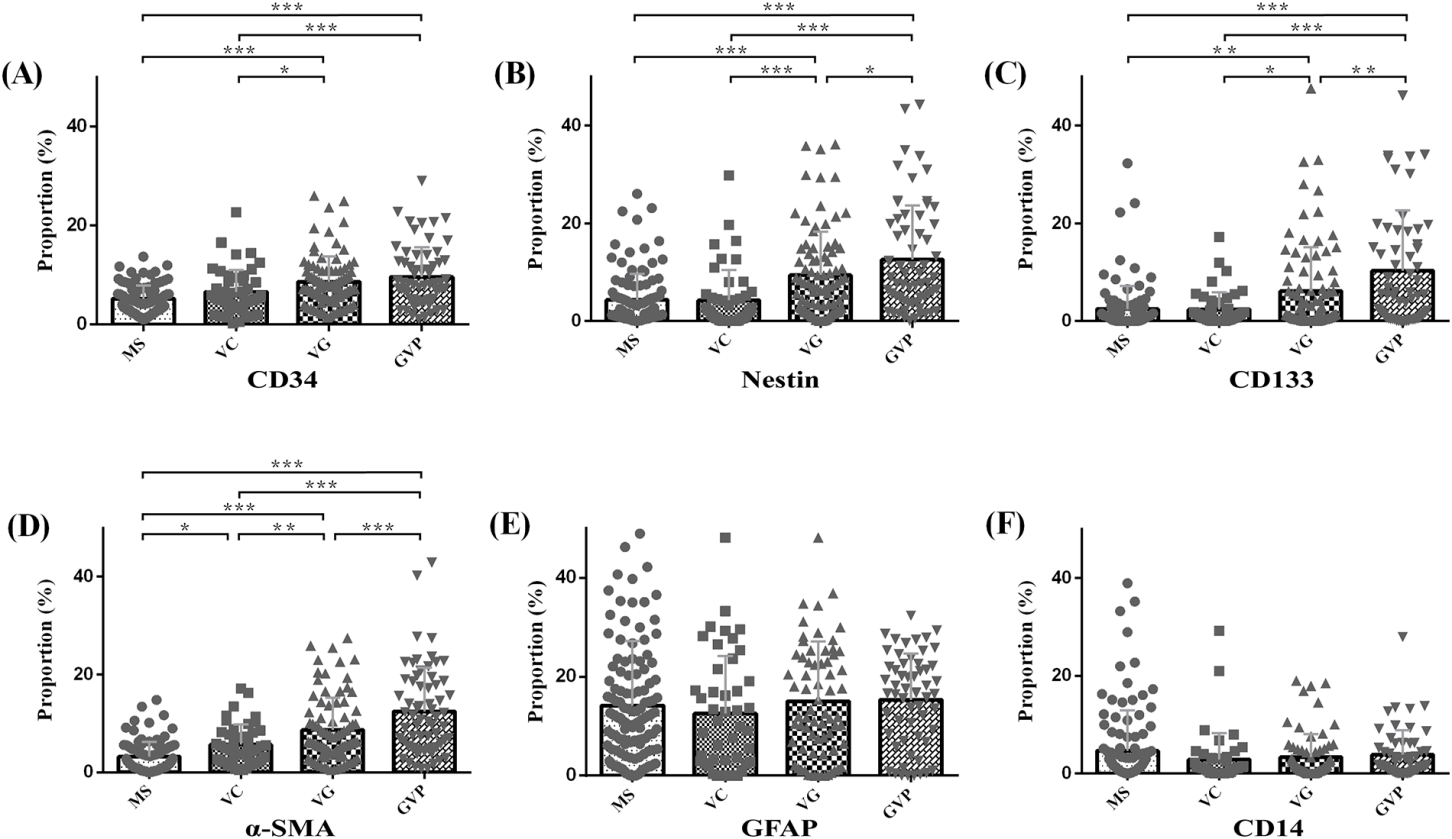
CD34, CD133, Nestin, α-SMA, GFAP and CD14 Area fraction in the different microvascular patterns. (A) CD34, in addition to MS&VC and VG&GVP are no significant difference (p>0.05), the other multiple comparisons are statistically significant. (B) (C) Nestin and CD133, the multiple comparisons are statistically significant besides MS&VC. (D) α-SMA, the pairwise Comparison of the α-SMA area fraction in the different microvascular patterns are statistically significant. (E) (F) GFAP and CD14, the pairwise Comparison of the area fraction aren’t statistically significant. Horizontal lines denote the mean and standard error of mean. * p<0.05; ** p<0.01; *** p<0.001.

**Fig 3:**
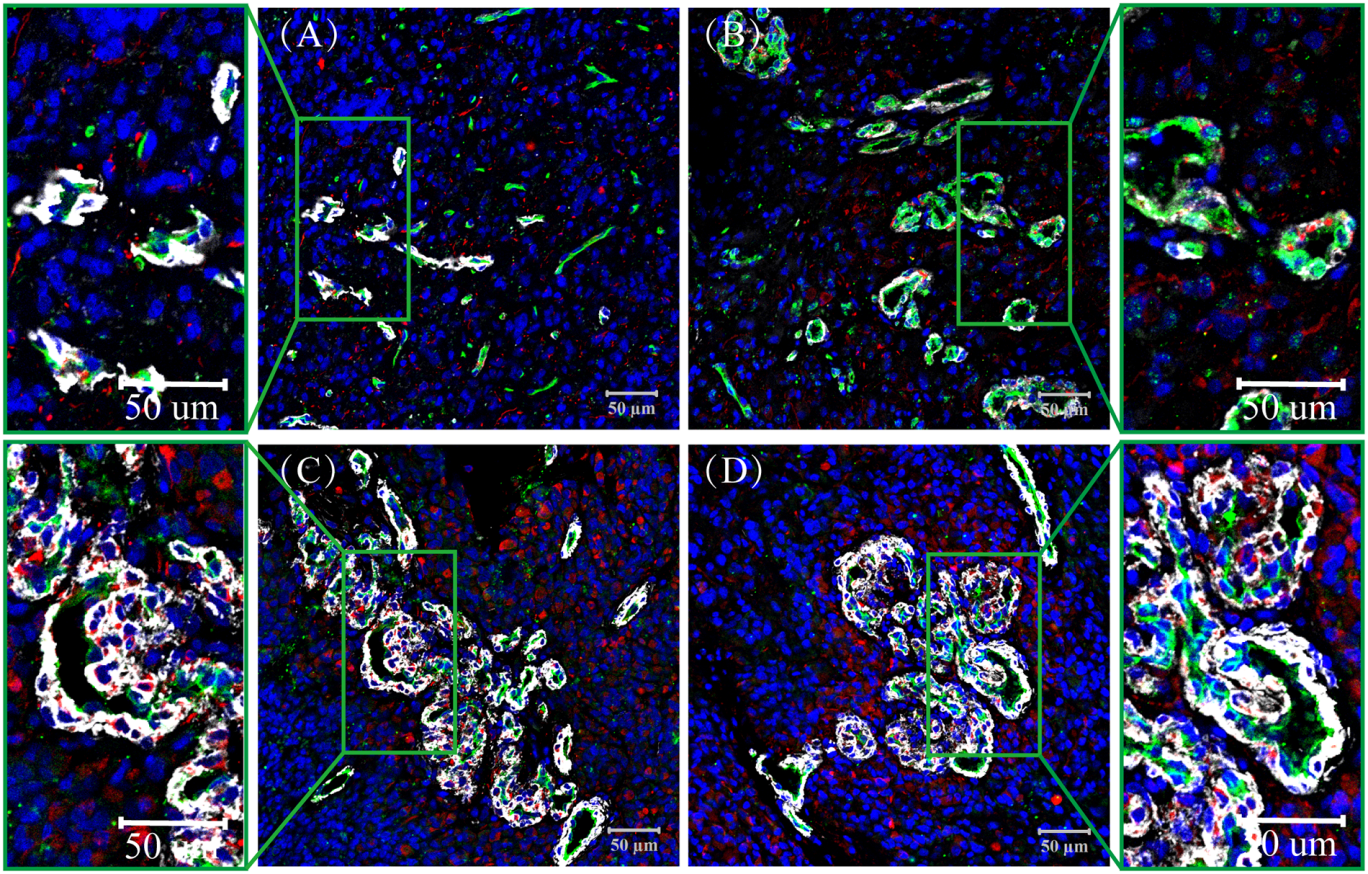
Nestin/α-SMA/CD34 Expression in Glioblastoma, α-SMA (Purple), Nestin (Red), CD34 (Green). (A) microvascular sprouting (MS), some of the CD31 positive blood vessels express α-SMA, but others do not; (B) vascular cluster (VC), many α-SMA positive cells surround VC; (C) vascular garland (VG), where most of the α-SMA positive cells accumulated around the blood vessels; (D) glomeruloid vascular proliferation (GVP), a large number of α-SMA positive cells were detected in the periphery of vessels, but fewer were present in the avascular area. Some Nestin positive cells co-expressed α-SMA. Nuclei are always counterstained with DAPI (Blue).

Four types of CD34-labeled microvessel formations in glioblastomas, as described by Birner et al. [21,22] and Chen et al. [20], were defined: (1) microvascular sprouting (MS), defined as delicate capillary-like microvessels resembling those seen during classic angiogenesis, distributed evenly throughout the major parts of vital tumor tissue (Fig 1A); (2) vascular cluster (VC), defined as distinct focal aggregations of vessels (≥ 3) without connective stroma (Fig 1B); (3) vascular garland (VG), defined as clustered vessels arranged in garland-like formation, with or without connective stroma, frequently located around necrotic tissue (Fig 1C); and (4) glomeruloid vascular proliferation (GVP), defined as clustered vessels (≥ 3) ensheathed by connective stroma (Fig 1D). Stained lumen or separate CD34-positive cells were considered a single countable microvessel, except for vessels with muscular walls.

### CD133 and Nestin positive cells in the perivascular niche

In glioblastoma samples, CD34 expression was observed in the cytoplasm of tumor vascular endothelial cells. The microvascular formations were varied and included single endothelium and tubular vessels in primary glioblastoma tissues (Fig 1 and S1 Fig). We classified CD34+ (green) vessels into four patterns. The percentage of CD34+ cells in MS and VC were lower than in VG and GVP (Table 2), and significant differences (P<0.05) were observed between them. We found that CD133 (purple) or Nestin (red) were not only expressed on GSCs but were also expressed in the brain capillaries on endothelial cells (Fig 1 and S1 Fig). We found that most of the CD133+ and Nestin+ cells exhibited a scattered distribution in MS and VC but that CD133+ and Nestin+ cells surrounded heterogeneous microvessels in most of the VG and GVP areas (Fig 1C and 1D). The area proportions of CD133+ cells were (2.50%, 0.00–32.33), (2.42%, 0.00–17.20), (6.15%, 0.00–47.62), and (10.36%, 0.00–51.11) in MS, VC, VG and GVP, respectively (Table 2). The area proportion of Nestin in MS (4.39%, 0.00–26.04) and VC (4.26%, 0.00–29.81) was significantly lower than in VG (9.41%, 0.01–36.20) and GVP (12.65%, 0.13–44.27) (Table 2, P < 0.05, Fig 2C). In addition, we found that a large amount of Nestin expression was located outside the microvessels and around ECs in VG and GVP (Fig 1C and 1D and S1 Table).

**Table 2:** Characteristics of different markers in glioblastoma microvascular patterns.

Microvascular parameters	MS	VC	VG	GVP
	Area Proportion (%) Median (range)	Area Proportion (%) Median (range)	Area Proportion (%) Median (range)	Area Proportion (%) Median (range)
**CD34**	5.23(1.01–13.72)	6.55(0.27–22.72)	8.65(1.50–26.01)	9.66(1.13–29.01)
**Nestin**	4.39(0.00–26.04)	4.26(0.00–29.81)	9.41(0.01–36.20)	12.65(0.13–44.27)
**CD133**	2.50(0.00–32.33)	2.42(0.00–17.20)	6.15(0.00–47.62)	10.36(0.00–51.11)
**α-SMA**	3.21(0.02–14.82)	5.66(0.42–17.13)	8.63(0.44–27.50)	12.47(0.75–42.92)
**GFAP**	14.17(0.01–63.78)	12.54(0.00–48.11)	15.04(0.00–52.41)	15.33(0.00–32.37)
**CD14**	4.70(0.00–50.73)	2.87(0.00–29.24)	3.36(0.00–19.04)	3.83(0.00–27.92)

Abbreviations: MS, microvascular sprouting; VC, vascular clusters; VG, vascular garlands; GVP, Glomeruloid Vascular Proliferation; one way ANOVA test.

### Nestin and α-SMA positive cells in the perivascular niche

In the present experiment, multiple markers were used to probe ECs, pericytes and GSCs. Immunofluorescence staining demonstrated that the pericyte marker a-SMA (purple) was located in the cell cytoplasm and that positive cells were located outside the microvessels and around ECs (Fig 3 and S2 Fig). We found that the thin-walled microvessels in MS were primarily composed of CD34 (green) positive ECs, with a few a-SMA positive cells (Fig 3A). Part of the thin-walled vascular cells showed no purple in MS (Fig 3A). With the increasing thickness of vessel walls, more purple stained cells appeared, enclosing the lining cells at the thick-wall, plexus and glomeruloid microvessels, especially in VG and GVP (Fig 3C and 3D). The results of Nestin/α-SMA/CD34 multiple immunofluorescence staining indicated that both the α-SMA+ cells and Nestin+ (red) cells accumulated around the CD34+ blood vessels. Moreover, some Nestin+ cells co-expressed α-SMA+ (Fig 3 and S2 Fig). The area proportion of α-SMA+ was (3.21%, 0.02–14.82), (5.66%, 0.42–17.13), (8.63%, 0.44–27.50), and (12.47%, 0.75–42.92) in MS, VC, VG and GVP, respectively, and there were significant differences between the groups (P < 0.05, Table 2, Fig 2B/2D and S1 Table).

### GFAP and CD14 positive cells in the perivascular niche

**Fig 4:**
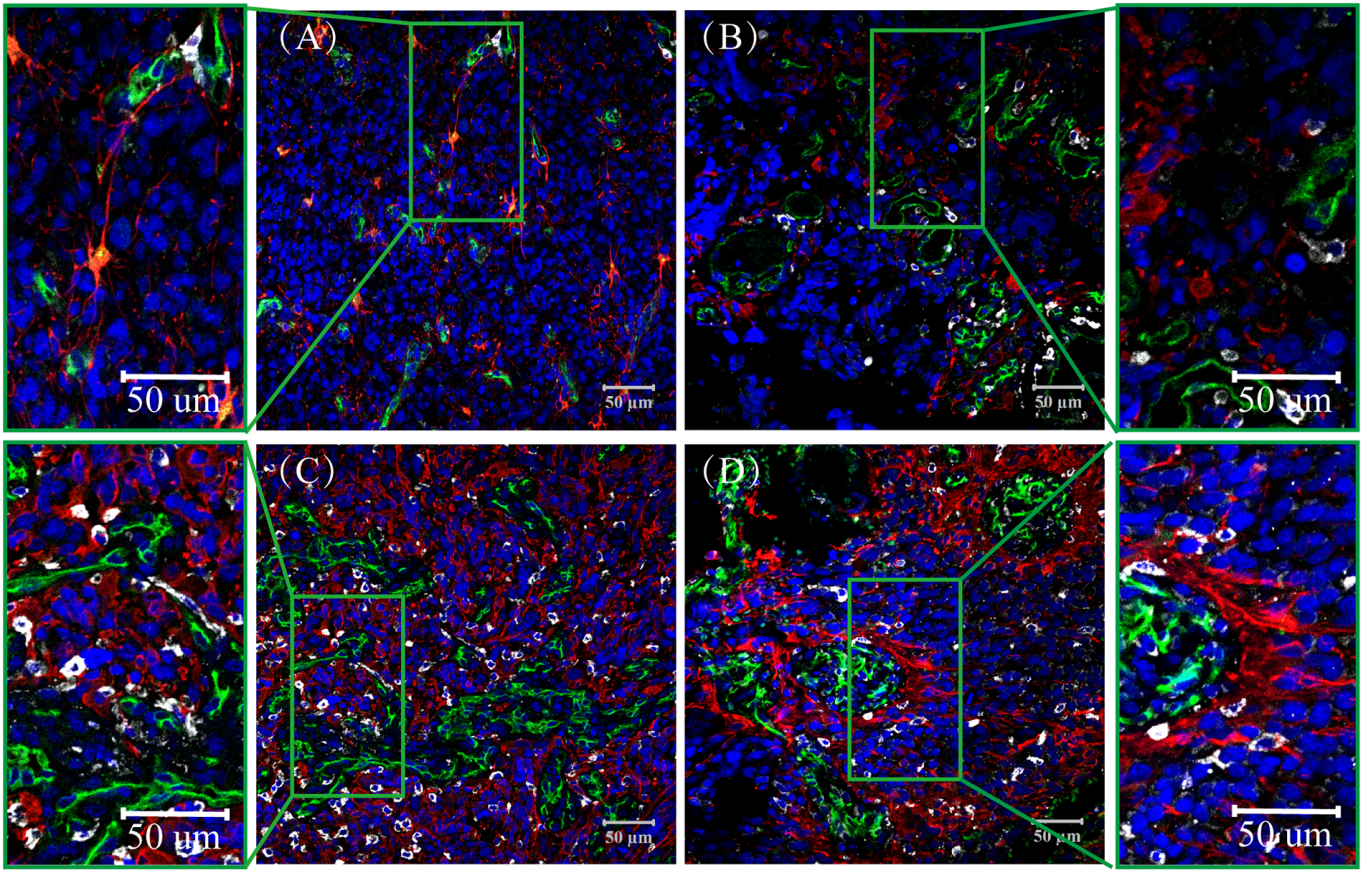
GFAP/CD14/CD34 Expression in Glioblastoma, CD14 (Purple), GFAP (Red), CD34 (Green). (A) microvascular sprouting (MS); (B) vascular cluster (VC); (C) vascular garland (VG); (D) glomeruloid vascular proliferation (GVP). Most of the GFAP and CD14 positive cells had a dispersive distribution in MS, VC, VG and GVP, and some GFAP positive cells accumulated around the blood vessels. Nuclei are Counterstained with DAPI (Blue).

The perivascular niche is not only important for the maintenance of stem cells, endothelial cells and pericytes, but it also contains an abundant subpopulation of astrocytic and macrophage infiltrates. In this study, we used GFAP (red) (Fig 4 and S3 Fig) as a marker for glial cells, and CD14 (purple) was utilized to label macrophages. In the differentiation of MVPNs, we observed that many GFAP+ cells were dispersed in MS, VC, VG and GVP. Unexpectedly, we observed that in some GVP, GFAP was abnormally distributed around the heterogeneous microvessels (Fig 4D). Most CD14 positive cells are distributed around vascular cells, and these cells are highly expressed in necrotic areas; moreover, a portion of the vessel lumen in VG and GVP microvascular patterns has CD14 positive cells (Fig 4 and S3 Fig). The area proportion of GFAP and CD14 was not significantly different among the four microvascular patterns (P > 0.05, Table 2, Fig 2E/2F and S1 Table).

**Fig 5:**
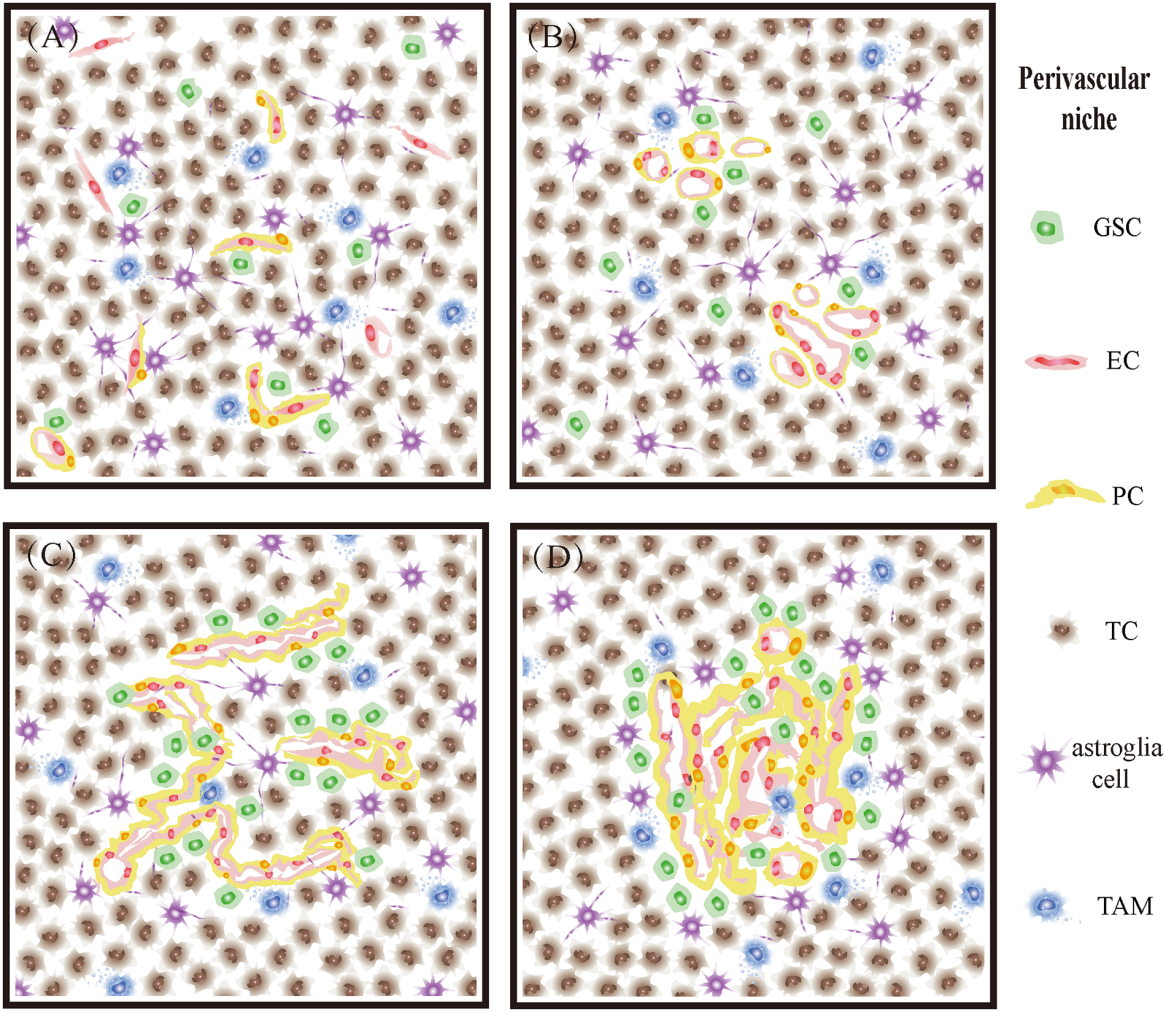
Characteristics of MVPNs though the classification of four different vascular patterns. (A) microvascular sprouting niche, (B) vascular cluster niche, (C) vascular garland niche, (D) glomeruloid vascular proliferation niche.

Based on the above analysis of quantitative and positional differences in tumor cells, endothelial cells, pericytes and tissue-specific components distributed in GBM, we describe the characteristics of MVPNs by classifying four different vascular patterns (Fig 5) and provide information on how to simulate the microenvironment of GBM more precisely. In the microvascular sprouting niche, substantial classic angiogenesis is uniformly distributed. Only a few GSCs, endothelial cells and pericytes were found, and they were scattered (Figs 4A and 5A). In the vascular cluster niche, more than three vessels were present, and few GSCs were around them. In addition, some of those vessels were surrounded by pericytes, and astrocytes showed a high genetic differentiation (Figs 4B and 5B). In the vascular garland niche vessels exhibited a cluster structure, which endothelial cells and pericytes grew irregularly. Most of the GSCs and astrocyte were located near the vessels (Figs 4C and 5C). In the glomeruloid vascular proliferation niche, endothelial cells gathered together and exhibited a similar structure as glomeruloid with different sizes. The abnormal proliferation of pericytes was observed around vessels, and GSCs showed a sandwich structure with the vessels (Figs 4D and 5D).

## Discussion

Glioblastoma is a highly vascularized malignant tumor with an obvious heterogeneity of vascular morphology. Birner et al. [21] and others and a preliminary study in this laboratory found a variety of vascular patterns and different expression patterns of different proteins that are relevant to the clinical prognosis of glioblastoma. This study investigated the relationships among different MVPs and glioma stem cells, pericytes, astrocytes, and macrophages in glioblastoma by analyzing four different MVPs of glioblastoma to further understand the structural features of the vascular niche and provide a basis for the precise in vitro simulation of the perivascular niche. In this study, the number of CD34 positive cells in MS and VC patterns was significantly lower than that in VG and GVP patterns. The expression status of CD133 and Nestin positive cells in these four vascular patterns was also observed. Most of the cells showed a scattered distribution in MS and VC patterns, whereas they were clustered or nested around the microvessels in VG and GVP patterns. These results are consistent with those of Zeppernick et al. [23]. With the increase in vascular heterogeneity, the positive rate is significantly increased. It may be hypothesized that GSCs maintain intimate contact with blood vessels and localize in the vicinity of blood vessels, which may promote their self-renewal and regenerative potential in VG and GVP, leading to resistance to therapy.

There was abundant α-SMA expression in the glomerular vascular plexus and glomerular vascular bundles [24,25], but most of the EC markers were only confined to the monolayer lining cells, which suggests that pericytes, not ECs, are the main cells that constitute the multiplicity of VG and GVP. More researchers have uncovered a similar phenomenon in higher pathologic grades of clear cell renal cell carcinoma and other malignant tumors [26]. Why there is such an abundance of pericytes, and where they come from? In fact, it must be considered a variety of sources, not only because they are of BM-MSC (Bone marrow-mesenchymal stem cells) origin, but also GSC can transdifferentiation into pericytes [27,28]. In our research, we found that in VG and GVP with the increase in vascular heterogeneity, α-SMA positive cells also exhibited abnormal proliferation, and most of the α-SMA positive cells co-expressed Nestin, which was GSCs marker especially evident in the VG and GVP patterns. Considering Cheng et al.’s reports most GSC can transdifferentiation into pericytes [29], we speculated that in addition to pericytes derived from BM-MSC, GSC transdifferentiate into pericytes seems to be a more likely and credible event in the perivascular niche. The same study provided evidence that the GSC can efficiently give rise to vessel-like tubular structures in a 3D Matrigel in vitro assay [30].

It is becoming increasingly clear that non-tumor cells play an important role in tumor progression and that the local induction of non-tumor cells is a characteristic feature of many tumors. GSCs secrete factors that support the growth of macrophages and induce the polarization of TAMs into the immunosuppressive M2 phenotype [31,32]. Accordingly, we observed GFAP/CD14 expression on the same tissue section to further understand the heterogeneity of the MVPN structure. The data analysis shows that there was no significant difference in the expression of GFAP and CD14 in the four different microvascular patterns. However, GFAP positive cells were abundant in most of the GBMs, and their distribution was more inordinate [33]. CD14 positive cells were concentrated in some tissues. Most of the necrotic areas over-expressed CD14, which may have been due to tumor cells being hypoxic when tumor angiogenesis was insufficient to maintain the growth of tumor cells, thus inducing tumor cells to secrete a series of inflammatory mediators and chemokines and causing microglia to gather around the tumor and mononuclear cells in the blood to migrate to the tumor site through the damaged blood-brain barrier. Within the perivascular niche, tumor associated macrophages (TAMs) were differentiated from microglia/macrophages, which resulted in a significant increase in CD14 expression in some regions and surrounding necrotic areas [31].

Given the complexity of the distribution of various cells in the perivascular niche, the statistical results showed that there were some differences between the vascular patterns after quantification was performed. It is noteworthy that the differences in CD133, Nestin, CD34 and α-SMA expression between MS and VG, MS and GVP, VC and VG, and VC and GVP were most obvious. Therefore, glioma stem cells and endothelial cells are the core of the perivascular niche and play crucial roles in the occurrence and development of GBM. The construction of an effective in vitro perivascular niche model is necessary to explore glioblastoma drug resistance and recurrence mechanisms and to search for a new effective target.

## Supporting information

S1 Protocol Protocol for cell stains. (DOCX)

S1 Table Characteristics of P-value among four microvascular patterns. (DOCX)

S1 Fig CD133/Nestin/CD34 Expression in Glioblastoma, CD133 (Purple), Nestin (Red), CD34 (Green). Nuclei are Counterstained with DAPI (Blue). (DOCX)

S2 Fig Nestin/α-SMA/CD34 Expression in Glioblastoma, α-SMA (Purple), Nestin (Red), CD34 (Green). Nuclei are Counterstained with DAPI (Blue). (DOCX)

S3 Fig GFAP/CD14/CD34 Expression in Glioblastoma, CD14 (Purple), GFAP (Red), CD34 (Green). Nuclei are counterstained with DAPI (Blue). (DOCX)

## Abbreviations

CD133: prominin1 (GBM stem cell marker)
CD14: Monocytes and macrophages antigen
CD34: hematopoietic progenitor cells and the small vessel endothelium of a variety of tissues (endothelial cell marker)
GFAP: Glial fibrillary acidic protein (astrocyte marker)
Nestin: a type VI intermediate filament protein (GBM stem cell marker)
α-SMA: alpha smooth muscle actin (perithelial cell marker)

## References

[1] Chepko G, Slack R, Carbott D, Khan S, Steadman L, Dickson RB. Differential alteration of stem and other cell populations in ducts and lobules of TGFalpha and c-Myc transgenic mouse mammary epithelium. Tissue Cell. 2005;37: 393–412. doi: 10.1016/j.tice.2005.06.005 16137731 10.1016/j.tice.2005.06.005

[2] Bao S, Wu Q, McLendon RE, Hao Y, Shi Q, Hjelmeland AB, et al Glioma stem cells promote radioresistance by preferential activation of the DNA damage response. Nature. 2006;444: 756–760. doi: 10.1038/nature05236 17051156 10.1038/nature05236

[3] Codrici E, Enciu AM, Popescu ID, Mihai S, Tanase C. Glioma stem cells and their microenvironments: providers of challenging therapeutic targets. Stem Cells Int. 2016;2016: 5728438 doi: 10.1155/2016/5728438 26977157 10.1155/2016/5728438 PMC4764748

[4] Brooks MD, Sengupta R, Snyder SC, Rubin JB. Hitting them where they live: targeting the glioblastoma perivascular stem cell niche. Curr Pathobiol Rep. 2013;1: 101–110. doi: 10.1007/s40139-013-0012-0 23766946 10.1007/s40139-013-0012-0 PMC3677798

[5] Hira VV, Verbovsek U, Breznik B, Srdic M, Novinec M, Kakar H, et al Cathepsin K cleavage of SDF-1alpha inhibits its chemotactic activity towards glioblastoma stem-like cells. Biochimica et biophysica acta. 2017;1864(3):594–603. doi: 10.1016/j.bbamcr.2016.12.021 28040478 10.1016/j.bbamcr.2016.12.021

[6] Pang YW, Feng J, Daltoe F, Fatscher R, Gentleman E, Gentleman MM, et al Perivascular stem cells at the tip of mouse incisors regulate tissue regeneration. J Bone Miner Res. 2016;31: 514–523. doi: 10.1002/jbmr.2717 26391094 10.1002/jbmr.2717 PMC5833940

[7] Schiffer D, Mellai M, Annovazzi L, Casalone C, Cassoni P. Tumor microenvironment: perivascular and perinecroticniches in gliomas In: Lichtor T, editor. Tumors of the central nervous system. Rijeka, Croatia: InTech Publishers; 2015 pp. 49–82.

[8] Oh M, Nor JE. The perivascular niche and self-renewal of stem cells. Front Physiol. 2015;6: 367 doi: 10.3389/fphys.2015.00367 26696901 10.3389/fphys.2015.00367 PMC4667083

[9] Louis DN, Ohgaki H, Wiestler OD, Cavenee WK, Burger PC, Jouvet A, et al The 2007 WHO classification of tumours of the central nervous system. Acta Neuropathol. 2007;114: 97–109. doi: 10.1007/s00401-007-0243-4 17618441 10.1007/s00401-007-0243-4 PMC1929165

[10] Stupp R, Mason WP, van den Bent MJ, Weller M, Fisher B, Taphoorn MJ, et al Radiotherapy plus concomitant and adjuvant temozolomide for glioblastoma. N Engl J Med. 2005;352: 987–996. doi: 10.1056/NEJMoa043330 15758009 10.1056/NEJMoa043330

[11] Hottinger AF, Pacheco P, Stupp R. Tumor treating fields: a novel treatment modality and its use in brain tumors. Neuro Oncol. 2016;18: 1338–1349. doi: 10.1093/neuonc/now182 27664860 10.1093/neuonc/now182 PMC5035531

[12] Wen PY, Kesari S. Malignant gliomas in adults. N Engl J Med. 2008;359: 492–507. doi: 10.1056/NEJMra0708126 18669428 10.1056/NEJMra0708126

[13] Ottone C, Krusche B, Whitby A, Clements M, Quadrato G, Pitulescu ME, et al Direct cell-cell contact with the vascular niche maintains quiescent neural stem cells. Nat Cell Biol. 2014;16: 1045–1056. doi: 10.1038/ncb3045 25283993 10.1038/ncb3045 PMC4298702

[14] Hayakawa Y, Ariyama H, Stancikova J, Sakitani K, Asfaha S, Renz BW, et al Mist1 expressing gastric stem cells maintain the normal and neoplastic gastric epithelium and are supported by a perivascular stem cell niche. Cancer Cell. 2015;28: 800–814. doi: 10.1016/j.ccell.2015.10.003 26585400 10.1016/j.ccell.2015.10.003 PMC4684751

[15] Lin CH, Lin XX, Lin L, Wang JM, Lin ZX, Lin JM. Development of LC—MS method for analysis of paclitaxel-inhibited growth and enhanced therapeutic response in human glioblastoma cells. Chinese Chemical Letters. 2015;26: 1225–1230.

[16] Zhu TS, Costello MA, Talsma CE, Flack CG, Crowley JG, Hamm LL, et al Endothelial cells create a stem cell niche in glioblastoma by providing NOTCH ligands that nurture self-renewal of cancer stem-like cells. Cancer Res. 2011;71: 6061–6072. doi: 10.1158/0008-5472.CAN-10-4269 21788346 10.1158/0008-5472.CAN-10-4269 PMC3355476

[17] Chen Q, Utech S, Chen D, Prodanovic R, Lin JM, Weitz DA. Controlled assembly of heterotypic cells in a core-shell scaffold: organ in a droplet. Lab Chip. 2016;16: 1346–1349. doi: 10.1039/c6lc00231e 26999495 10.1039/c6lc00231e PMC4829496

[18] Filatova A, Acker T, Garvalov BK. The cancer stem cell niche(s): the crosstalk between glioma stem cells and their microenvironment. Biochim Biophys Acta. 2013;1830: 2496–2508. doi: 10.1016/j.bbagen.2012.10.008 23079585 10.1016/j.bbagen.2012.10.008

[19] Paez-Ribes M, Allen E, Hudock J, Takeda T, Okuyama H, Vinals F, et al Antiangiogenic therapy elicits malignant progression of tumors to increased local invasion and distant metastasis. Cancer Cell. 2009;15: 220–231. doi: 10.1016/j.ccr.2009.01.027 19249680 10.1016/j.ccr.2009.01.027 PMC2874829

[20] Chen L, Lin ZX, Lin GS, Zhou CF, Chen YP, Wang XF, et al Classification of microvascular patterns via cluster analysis reveals their prognostic significance in glioblastoma. Hum Pathol. 2015;46: 120–128. doi: 10.1016/j.humpath.2014.10.002 25455996 10.1016/j.humpath.2014.10.002

[21] Birner P, Piribauer M, Fischer I, Gatterbauer B, Marosi C, Ambros PF, et al Vascular patterns in glioblastoma influence clinical outcome and associate with variable expression of angiogenic proteins: evidence for distinct angiogenic subtypes. Brain Pathol. 2003;13: 133–143. 12744467 10.1111/j.1750-3639.2003.tb00013.x PMC8095831

[22] Louveau A, Smirnov I, Keyes TJ, Eccles JD, Rouhani SJ, Peske JD, et al Structural and functional features of central nervous system lymphatic vessels. Nature. 2015;523: 337–341. doi: 10.1038/nature14432 26030524 10.1038/nature14432 PMC4506234

[23] Zeppernick F, Ahmadi R, Campos B, Dictus C, Helmke BM, Becker N, et al Stem cell marker CD133 affects clinical outcome in glioma patients. Clin Cancer Res. 2008;14: 123–129. doi: 10.1158/1078-0432.CCR-07-0932 18172261 10.1158/1078-0432.CCR-07-0932

[24] Sun H, Guo D, Su Y, Yu D, Wang Q, Wang T, et al Hyperplasia of pericytes is one of the main characteristics of microvascular architecture in malignant glioma. PLoS One. 2014;9: e114246 doi: 10.1371/journal.pone.0114246 25478951 10.1371/journal.pone.0114246 PMC4257691

[25] Svensson A, Ozen I, Genove G, Paul G, Bengzon J. Endogenous brain pericytes are widely activated and contribute to mouse glioma microvasculature. PLoS One. 2015;10: e0123553 doi: 10.1371/journal.pone.0123553 25875288 10.1371/journal.pone.0123553 PMC4395339

[26] Cao Y, Zhang ZL, Zhou M, Elson P, Rini B, Aydin H, et al Pericyte coverage of differentiated vessels inside tumor vasculature is an independent unfavorable prognostic factor for patients with clear cell renal cell carcinoma. Cancer. 2013;119: 313–324. doi: 10.1002/cncr.27746 22811049 10.1002/cncr.27746

[27] Bexell D, Gunnarsson S, Tormin A, Darabi A, Gisselsson D, Roybon L, et al Bone Marrow Multipotent Mesenchymal Stroma Cells Act as Pericyte-like Migratory Vehicles in Experimental Gliomas. Molecular Therapy. 2009;17:183–90. doi: 10.1038/mt.2008.229 18985030 10.1038/mt.2008.229 PMC2834971

[28] Barcellos-de-Souza P, Gori V, Bambi F, Chiarugi P. Tumor microenvironment: bone marrow-mesenchymal stem cells as key players. Biochimica et biophysica acta. 2013;1836: 321–35. doi: 10.1016/j.bbcan.2013.10.004 24183942 10.1016/j.bbcan.2013.10.004

[29] Cheng L, Huang Z, Zhou W, Wu Q, Donnola S, Liu JK, et al Glioblastoma stem cells generate vascular pericytes to support vessel function and tumor growth. Cell. 2013;153: 139–152. doi: 10.1016/j.cell.2013.02.021 23540695 10.1016/j.cell.2013.02.021 PMC3638263

[30] Birbrair A, Zhang T, Wang ZM, Messi ML, Olson JD, Mintz A, et al Type-2 pericytes participate in normal and tumoral angiogenesis. Am J Physiol Cell Physiol. 2014;307: C25–38. doi: 10.1152/ajpcell.00084.2014 24788248 10.1152/ajpcell.00084.2014 PMC4080181

[31] Zhou W, Ke SQ, Huang Z, Flavahan W, Fang X, Paul J, et al Periostin secreted by glioblastoma stem cells recruits M2 tumour-associated macrophages and promotes malignant growth. Nat Cell Biol. 2015;17: 170–182. doi: 10.1038/ncb3090 25580734 10.1038/ncb3090 PMC4312504

[32] Lapa C, Linsenmann T, Luckerath K, Samnick S, Herrmann K, Stoffer C, et al Tumor-associated macrophages in glioblastoma multiforme-a suitable target for somatostatin receptor-based imaging and therapy? PLoS One. 2015;10: e0122269 doi: 10.1371/journal.pone.0122269 25807228 10.1371/journal.pone.0122269 PMC4373835

[33] Guichet PO, Guelfi S, Ripoll C, Teigell M, Sabourin JC, Bauchet L, et al Asymmetric distribution of GFAP in glioma multipotent cells. PLoS One. 2016;11: e0151274 doi: 10.1371/journal.pone.0151274 26953813 10.1371/journal.pone.0151274 PMC4783030

