# Supplementary material for: The pathological structure of the perivascular niche in different microvascular patterns of glioblastoma
Source: PLoS One. 2017 Aug 3;12(8):e0182183. doi: 10.1371/journal.pone.0182183 (PMC5542434; doi:10.1371/journal.pone.0182183)
Supplement: S1 Protocol — (DOCX) [file pone.0182183.s001.docx]

**Protocols for cell stains**

1. Sections were fixed in 10% Neutral Buffered Formalin (NBF), processed with the Leica ASP6025 tissue processor (Leica Microsystems, Germany), embedded in paraffin and cut into several continuous sections at a thickness of 4 μm.
2. Sections were deparaffinized in xylene, dehydrated in graded alcohol for the stated time:

(1) Xylene 3 changes, 5 min each

(2) Absolute ethanol two changes, 5 min each

(3) Ninety percent ethanol one change for 3 min

(4) Seventy percent ethanol one change for 3 min

(5) Fifty percent ethanol one change for 3 min

1. Place slides in water and leave container in a 37°C water bath for 3 min.
2. Antigens were retrieved with All-purpose Powerful Antigen Retrieval Solution (1:10, Beyotime, P0088, China) in a beaker containing the slides. Place beaker on a hot plate and boil for 25 min, then place the beaker on the counter and cool for 30 min.
3. Place sections in cool 0.1M PBS. Then place slides in a humid chamber.
4. Shake off excess buffer and slides were blocked for 30 minutes with QuickBlock™ Blocking Buffer (Beyotime, P0220, China) at room temperature.
5. Shake off normal serum and sections were first incubated with the goat anti-human CD34 (1:100, Santa Cruz, SC-7045, USA) for 4°C overnight (approximately 16 h).
6. Followed next day wash slides 3 times with PBS and visualized with Alexa Fluor® 488 incubate at room temperature for 60 min in the dark.
7. Wash slides 3 times with PBS for 5 min each and apply serum blocking buffer for 30 min at room temperature.
8. Shake off normal serum and apply mouse anti-Nestin antibody (1:150, Abcam, ab22035, UK) for 2 h at room temperature (mouse anti-GFAP, 1:100, ZSGB-BIO, ZM-0118, China) .The dilution of the antibody and the incubation time should be determined empirically.
9. Wash slides 3 times with PBS and apply Alexa Fluor® 555 incubate at room temperature for 60 min in the dark.
10. Repeat step 9, shake off excess buffer and apply the rabbit anti-CD133 antibody (1:100, biorbyt, orb99113, UK) incubate at room temperature for 2 h (anti-α-SMA, 1:200, Abcam, ab5694, UK; anti-CD14, 1:100, Abcam, ab133335, UK).
11. Wash slides 3 times with PBS and apply Alexa Fluor® 647 incubate at room temperature for 60 min in the dark.
12. Wash slides 3 times with PBS for 5 min each. Slides were counterstained with DAPI (4‘,6-diamidino-2-phenylindole Beyotime C1002, China) at 5 mg/ml in PBS for 10 minutes.
13. Repeat wash the slides and mounted with Antifade Mounting Medium kept at -20 °C.
