# Supplementary material for: The pathological structure of the perivascular niche in different microvascular patterns of glioblastoma
Source: PLoS One. 2017 Aug 3;12(8):e0182183. doi: 10.1371/journal.pone.0182183 (PMC5542434; doi:10.1371/journal.pone.0182183)
Supplement: S1 Table — (DOCX) [file pone.0182183.s002.docx]

S1 Table. Characteristics of P-value among four microvascular patterns

| Microvascular formations | P-value | | | | | |
| --- | --- | --- | --- | --- | --- | --- |
|  | MS&VC | MS&VG | MS&GVP | VC&VG | VC&GVP | VG&GVP |
| CD34 | 0.097 | 0.000 | 0.000 | 0.011 | 0.000 | 0.181 |
| Nestin | 0.926 | 0.000 | 0.000 | 0.000 | 0.000 | 0.015 |
| CD133 | 0.958 | 0.002 | 0.000 | 0.011 | 0.000 | 0.002 |
| α-SMA | 0.020 | 0.000 | 0.000 | 0.007 | 0.000 | 0.000 |
| GFAP | 0.433 | 0.622 | 0.544 | 0.277 | 0.246 | 0.896 |
| CD14 | 0.108 | 0.169 | 0.408 | 0.698 | 0.463 | 0.685 |

Abbreviations: MS, microvascular sprouting; VC, vascular clusters; VG, vascular garlands; GVP, Glomeruloid Vascular Proliferation;
