# Supplementary material for: The pathological structure of the perivascular niche in different microvascular patterns of glioblastoma
Source: PLoS One. 2017 Aug 3;12(8):e0182183. doi: 10.1371/journal.pone.0182183 (PMC5542434; doi:10.1371/journal.pone.0182183)
Supplement: S1 Fig — (DOCX) [file pone.0182183.s003.docx]

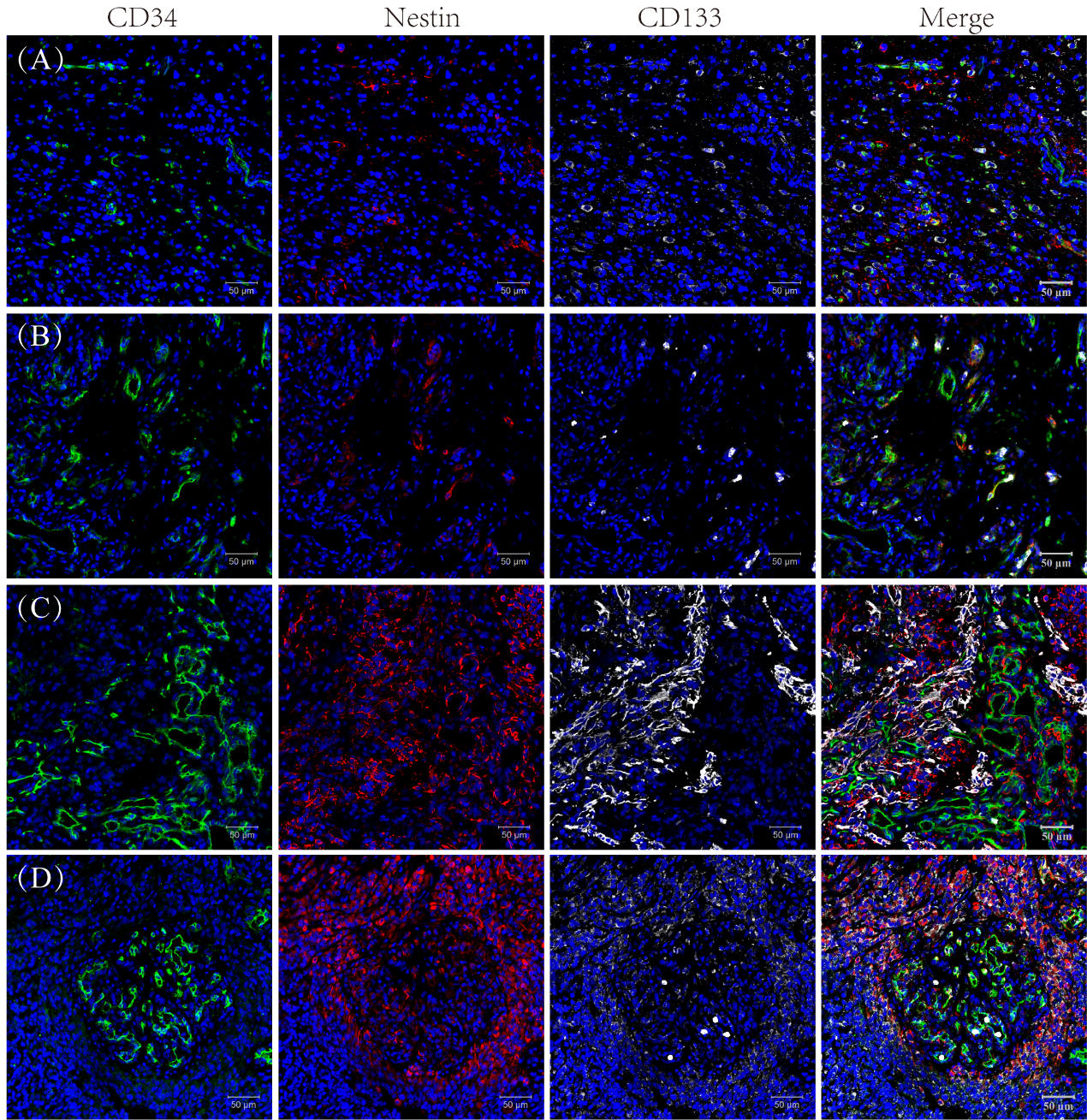


S1 Fig CD133 / Nestin/CD34 Expression in Glioblastoma, CD133 (Purple), Nestin (Red), CD34 (Green). Nuclei are Counterstained with DAPI (Blue).
